# A new species and a new record of the Southeast Asian millipede genus *Antheromorpha* Jeekel, 1968 (Polydesmida, Paradoxosomatidae) from Vietnam

**DOI:** 10.3897/zookeys.832.32596

**Published:** 2019-03-19

**Authors:** Natdanai Likhitrakarn, Sergei I. Golovatch, Irina Semenyuk, Somsak Panha

**Affiliations:** 1 Division of Plant Protection, Faculty of Agricultural Production, Maejo University, Chiang Mai 50290, Thailand Maejo University Chiang Mai Thailand; 2 AN Severtsov Institute for Problems of Ecology and Evolution, Russian Academy of Sciences, Leninsky pr. 33, Moscow 119071, Russia AN Severtsov Institute for Problems of Ecology and Evolution, Russian Academy of Sciences Moscow Russia; 3 Joint Russian-Vietnamese Tropical Center, Street 3 Thang 2, Q10, Ho Chi Minh City, Vietnam Joint Russian-Vietnamese Tropical Center Ho Chi Minh City Vietnam; 4 Animal Systematics Research Unit, Department of Biology, Faculty of Science, Chulalongkorn University, Bangkok, 10330, Thailand Chulalongkorn University Bangkok Thailand

**Keywords:** *
Antheromorpha
*, millipede, new species, Orthomorphini, taxonomy, Vietnam

## Abstract

*Antheromorphanguyeni***sp. n.** is described and illustrated from Kon Ka Kinh National Park, southern Vietnam. The new species is distinguished by a peculiar colour pattern showing a uniformly black-brown body contrasting with yellow-brown paraterga and epiproct, as well as in the pointed gonopodal process being unusually short, only approximately half as long as the solenophore. In addition, an identification key to all 13 presently known species, all mapped, is given. A new record of *A.festiva* is provided from southern Vietnam.

## Introduction

The Southeast Asian millipede genus *Antheromorpha* Jeekel, 1968 was established to replace *Brachytropis* Silvestri, 1896 which had been preoccupied by *Brachytropis* Fieber, 1858, a genus of Hemiptera (Jeekel 1968). It was later redefined, especially against the similarly large-bodied, even more species-rich, and mostly sympatric genus *Orthomorpha* Bollman, 1893, with some Burmese species revised and a few new synonymies proposed based on type material (Jeekel 1980). *Antheromorpha* has since been reviewed and rediagnosed, with even more synonymies established, and most species likewise redescribed, based both on type and fresh material (Likhitrakarn et al. 2016).

It was Attems (1937) who described the first member of this genus from Vietnam, *Orthomorphaharpaga* Attems, 1937, from Hon Ba Mountain in the south-central part of the country. Jeekel (1968, 1980) correctly transferred it to *Antheromorpha*. Likhitrakarn et al. (2016) have since redescribed this species from the types, while Golovatch and Semenyuk (2018) have freshly documented and illustrated it from Kon Ka Kinh National Park, Gia Lai Province, central highlands of Vietnam. Nguyen et al. (2018) have recently reviewed *Antheromorpha* in the scope of the Vietnamese fauna, described a new species, *A.pumatensis* Nguyen, Nguyen & Le, 2018 from Nghe An Province, north-central Vietnam, and provided additional records of *A.paviei* (Brölemann, 1896) from Ba Na National Park, Da Nang Province, and of *A.festiva* (Brölemann, 1896) from Kien Giang, Dak Lak and Tay Ninh provinces (Fig. 5).

The present paper adds to the record another new species of this genus from Vietnam, the fifth to be found in that country. This brings the diversity of *Antheromorpha* to a total of 13 recognised species that occur only in mainland Southeast Asia: Myanmar (6 species: *A.bistriata* (Pocock, 1895), *A.comotti* (Pocock, 1895), *A.mediovirgata* (Carl, 1941), *A.minlana* (Pocock, 1895), *A.miranda* (Pocock, 1895), *A.pardalis* (Pocock, 1895)), Vietnam (5 species: *A.festiva* (Brölemann, 1896), *A.harpaga* (Attems, 1937), *A.nguyeni* sp. n., *A.paviei* (Brölemann, 1896), *A.pumatensis* Nguyen, Nguyen & Le, 2018), and Thailand (3 species: *A.rosea* Golovatch, 2013, *A.festiva* (Brölemann, 1896), *A.uncinata* (Attems, 1931)). Southern China (*A.rosea* Golovatch, 2013), Laos (*A.paviei* (Brölemann, 1896)), and Western Malaysia (*A.festiva* (Brölemann, 1896)) currently support only a single species each (Fig. 5). Because a full catalogue of all previously described species and their distributions are available elsewhere (Likhitrakarn et al. 2016; Nguyen et al. 2018), we simply list them below in alphabetic order:

*Antheromorphabistriata* (Pocock, 1895)

*Antheromorphacomotti* (Pocock, 1895)

*Antheromorphafestiva* (Brölemann, 1896)

*Antheromorphaharpaga* (Attems, 1937)

*Antheromorphamediovirgata* (Carl, 1941)

*Antheromorphaminlana* (Pocock, 1895)

*Antheromorphamiranda* (Pocock, 1895)

*Antheromorphanguyeni* sp. n.

*Antheromorphapardalis* (Pocock, 1895)

*Antheromorphapaviei* (Brölemann, 1896)

*Antheromorphapumatensis* Nguyen, Nguyen & Le, 2018

*Antheromorpharosea* Golovatch, 2013

*Antheromorphauncinata* (Attems, 1931)

## Materials and methods

The material documented below was collected by one of us (Irina Semenyuk abbreviated IS), according to Agreements 432/TCLN-BTTN and 142/SNgV-VP between the Kon Ka Kinh National Park and the Joint Russian-Vietnamese Tropical Center, as part of IS’s research project on the diversity, biology, and ecology of millipedes in Vietnam.

Live animals were photographed in their habitats using a Canon 70D digital camera with a Canon PowerShot A4000IS 16.0 MP Digital Camera. Specimens were preserved in 75% ethanol, and morphological investigations were carried out in the laboratory using an Olympus stereo microscope. Scanning electron micrographs (SEM) of gold-coated gonopods were taken using a JEOL, JSM–5410 LV microscope. Specimens were also photographed and the images stacked in the laboratory using the “CellD” automontage software of the Olympus Soft Imaging Solution package, while the gonopods of a paratype were dissected and illustrated under Euromex iScope microscopes. Almost all material is housed in the Zoological Museum, State University of Moscow (ZMUM), Russia, with one paratype donated to the collection of Chulalongkorn University’s Museum of Zoology (CUMZ), Bangkok, Thailand.

In the synonomy section given below, **D** stands for a description or descriptive notes, **R** for a new record or records, while **M** for a mere mention. Other abbreviations are:

**d** gonopod process **d**, a distinct lobe on middle part of lamina lateralis, seen in mesal view

**m** gonopod process **m**, a small lower lobe on distal part of gonopod, clearly seen in mesal view

**s** lateral sulcus, a distinct sulcus distally on femur, visible on femur in lateral view

**sl** solenomere, a usually long, flagellum-like appendage, originating on base of solenophore

**sph** solenophore (= tibiotarsus), apical part of telopodite, consisting of lamina lateralis and lamina medialis

**v** gonopod process **v**, a small upper lobe on distal part of gonopod, clearly seen in mesal view

**CUMZ** Chulalongkorn University Museum of Zoology, Bangkok, Thailand

**ZMUM** Zoological Museum, University of Moscow, Russia

a.s.l. above sea level

ca. approximately, around (circa)

The Animal Care and Use Protocol Review No. 1723018 was applied.

Coordinates and elevation were recorded by Garmin GPSMAP 60 CSx and Garmin eTrex 30 using the WGS84 datum and subsequently double-checked with Google Earth.

## Taxonomy

### Family Paradoxosomatidae Daday, 1889

#### Subfamily Paradoxosomatinae Daday, 1889

##### Tribe Orthomorphini Brölemann, 1916

###### Genus *Antheromorpha* Jeekel, 1968

####### Antheromorpha festiva

Taxon classificationAnimaliaPolydesmidaParadoxosomatidae

(Brölemann, 1896)

Fig. 1

Orthomorpha festiva Brölemann, 1896: 1 (D).
Orthomorpha festiva : Attems 1898: 339 (M); 1914: 194 (D); 1930: 131 (D); Brölemann 1904: 4 (D, R).
Orthomorpha (Orthomorpha) festiva : Attems 1936: 199 (M); 1937: 60 (D).
“Orthomorpha” festiva Jeekel, 1963: 269 (M).
Antheromorpha festiva Jeekel, 1968: 57 (M); 1980: 85 (M); Golovatch 1983: 181 (M); Enghoff et al. 2004: 37 (M); Enghoff 2005: 95 (R); Nguyen and Sierwald 2013: 1234 (M); Likhitrakarn et al. 2016: 45 (D); Nguyen et al. 2018: 98 (D, R).

######## Material examined.

3 ♂ (ZMUM), Vietnam, Dong Nai Province, Cat Tien National Park, 11°25'37"N, 107°25'39"E, 140 m a.s.l., secondary monsoon lowland forest with dominating *Lagerstroemiacalyculata*, on forest floor, 10.V.2015; 1 ♂ (ZMUM), same locality, on ground road in grasslands, 11°24'20"N, 107°24'17"E, 120 m a.s.l., 14.XI.2014, all leg. I Semenyuk.

######## Remarks.

**Figure 1.**
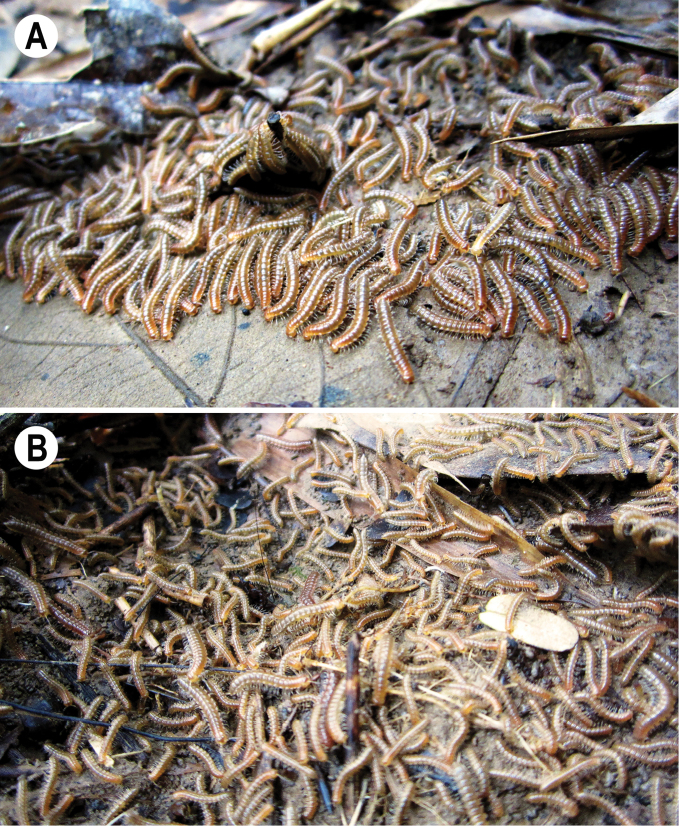
Swarming later instars of *Antheromorphafestiva* (Brölemann, 1896) in Cat Tien National Park. Photographs by I Semenyuk.

The new specimens fully agree with the detailed and beautifully illustrated redescriptions of the species as given by Brölemann (1904) and Likhitrakarn et al. (2016). This is the first formal record of *A.festiva* in Cat Tien National Park. In that park, *A.festiva* shows a pronounced seasonal rhythm. According to several years of observation by one of us (IS), juveniles of different stages (from the 4^th^ to the last instar) start swarming in the autumn, just before the dry season. Swarms contain hundreds of millipedes slowly moving around and feeding. One swarm patch usually contains mainly same-age individuals (Fig. 1). Even though swarms of different instars may appear next to each other, they normally do not mix. Patches of swarming are usually localized in secondary forest with dominating bamboo and in grasslands. Swarms of individuals of the latest instars are looser than those of earlier stages. Adults appear during the same season, but do not swarm. In the dry season, the abundance of *A.festiva* abruptly declines and then, in the early rainy season in summer, it gradually grows again to abruptly stop in the middle of the rainy season.

####### Antheromorpha nguyeni sp. n.

Taxon classificationAnimaliaPolydesmidaParadoxosomatidae

http://zoobank.org/98A9247E-F3B0-4D4A-B1E2-08DF101ABF4F

Figs 2 , 3 , 4

######## Type material.

Holotype. ♂ (ZMUM), Vietnam, Gia Lai Province, Kon Ka Kinh National Park, 14°13'08"N, 108°19'31"E, 1200 m a.s.l., tropical forest with *Lithocarpus* spp. abundant on hill slopes, on forest floor, daytime, 24.V.2017, leg. I Semenyuk.

***Paratypes.*** 1 ♂ (ZMUM), same locality, together with holotype; 1 ♂ (ZMUM), same locality as holotype, but on top of a hill with cloud forest, 14°13'12"N, 108°19'54"E, 1500 m a.s.l., on log, daytime, 26.V.2017; 1 ♂ (CUMZ), same locality as holotype, broadleaved tropical forest in river valley, on log, 800 m a.s.l., 14°12'46"N, 108°18'55"E, night time 22.V.2017, all leg. I Semenyuk.

######## Name.

To honour Nguyen Duc Anh, the leading Vietnamese myriapologist.

######## Diagnosis.

Differs from congeners mainly in the colour pattern (a uniformly black-brown body with contrasting yellow-brown paraterga and epiproct), as well as in gonopod process **d** being unusually short, approximately half as long as the solenophore.

######## Description.

Length of holotype 41.5 mm, width of midbody pro- and metazonae 3.2 and 4.7 mm, respectively. Paratypes 39.5–42.5 mm long, 2.9–3.8 and 4.5–5.1 mm wide on midbody pro- and metazonae, respectively (♂).

**Figure 2.**
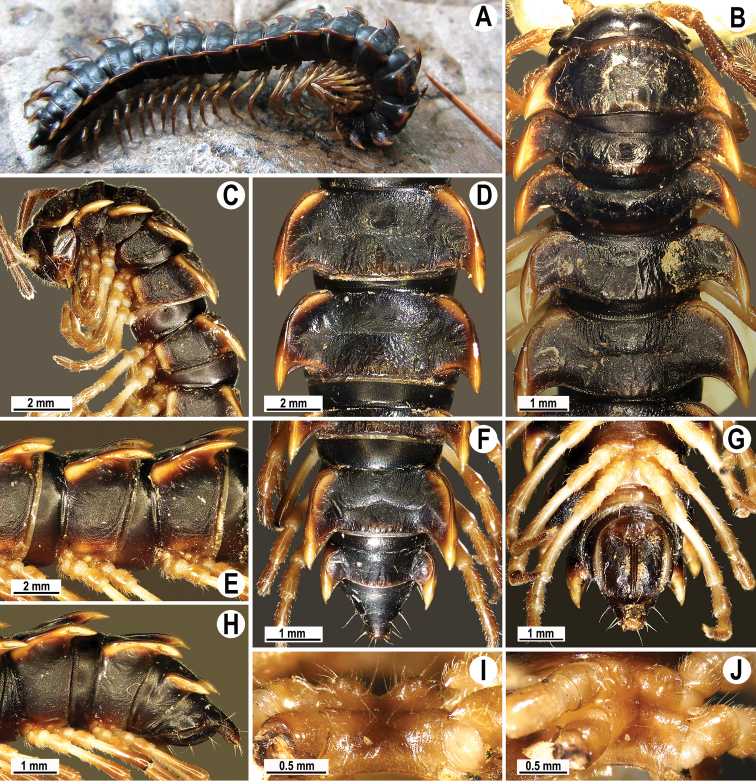
*Antheromorphanguyeni* sp. n., ♂ holotype. **A** habitus, live coloration; **B, C** anterior part of body, dorsal and lateral views, respectively **D, E** segments 10 and 11, dorsal and lateral views, respectively **F, G, H** posterior part of body, dorsal, ventral and lateral views, respectively **I, J** sternal cones between coxae 4, subcaudal and sublateral views, respectively.

Colouration of live animals blackish (Fig. 2A), edges of paraterga dark to light brown; antennae dark brownish, legs and venter dark to light brown (Fig. 2A); colouration in alcohol, after one-year-long preservation, faded to dark brownish (Fig. 2B–H), edge of paraterga faded to brownish or pale brown, antennae legs and venter light brown to pale yellowish (Fig. 2B–J).

Clypeolabral region sparsely setose; epicranial suture distinct. Antennae long (Fig. 2C), extending behind metaterga 3 when stretched dorsally (♂). In width, head < segment 4 < 3 < collum < segment 2 < 5 < 6–17 (♂), body gently and gradually tapering thereafter. Collum with three transverse rows of setae: 3+3 in anterior, 2+2 in intermediate, and 3+3 in posterior row; paraterga very broad (Fig. 2B), slightly upturned, anterior edge angular, lateral edge smooth, caudal corner almost or fully pointed, extending behind rear tergal margin (Fig. 2B, C).

Tegument generally smooth and poorly shining, prozonae finely shagreened, metaterga leathery and rugulose (Fig. 2A, B, D, F), surface below paraterga finely microgranulate (Fig. 2C, E, H). Postcollum metaterga with two transverse rows of setae traceable at least as insertion points when setae broken off: 2+2 in anterior (pre-sulcus) and 3+3 in posterior (post-sulcus) row. Tergal setae simple, slender, ca. 1/3 of metatergal length. Axial line visible only on metazonae, starting with collum. Paraterga very strongly developed (Fig. 1B–H), mostly clearly upturned above dorsum; only paraterga 2–4 slightly upturned, all lying below dorsum, set at ca. upper 1/3 (segments 2 and 3) or 1/4 (segment 4 and following ones) of body height, moderately enlarged in lateral view on pore-bearing segments, thinner on poreless ones; anterior edge broadly rounded and narrowly bordered, fused to callus; calluses delimited by a sulcus only dorsally on segments 2–4, following segments delimited by a sulcus both dorsally, and, albeit more poorly so, ventrally, in dorsal view narrower on poreless segments than on pore-bearing ones; lateral edge without incisions, caudal corners narrowly rounded to fully pointed, always extending behind rear tergal margin, posterior edge oblique to clearly concave, especially well so in segments 16–19 (Fig. 1B, D, F). Ozopores evident, lateral, lying in an ovoid groove at ca. 1/4 of metatergal length in front of caudal corner. Transverse sulcus usually distinct (Fig. 2B, D, F), complete on metaterga 5–18, shallow, reaching the bases of paraterga, very faintly beaded at bottom, incomplete and nearly wanting on segments 5 and 19. Stricture between pro- and metazona rather wide, deep, beaded at bottom down to base of paraterga (Fig. 2B–D, F). Pleurosternal carinae complete crests with a sharp caudal tooth on segments 2–4, thereafter crests bulged anteriorly and with a small, sharp, caudal tooth on segments 5–7, with only a small, sharp, caudal tooth on segments 8–10, and a very small denticle on segments 11–16 (Fig. 2C, E, H). Epiproct (Fig. 2H) long, stout, conical, flattened dorsoventrally, with two evident, caudoventrally curved, apical papillae; tip subtruncate; pre-apical papillae small, but evident, lying rather close to tip. Hypoproct subtriangular, caudal margin round, setiferous knobs at caudal edge evident and clearly separated.

Sterna sparsely setose, shining, cross-impressions shallow, without modifications; but with two rounded, low, fully separated, setose cones between ♂ coxae 4 (Fig. 2I, J). A conspicuous ridge present in front of gonopod aperture. Legs moderately long and slender, midbody ones ca. 1.2–1.5 times as long as body height, prefemora without modifications, ♂ tarsal brushes present until segment 17.

**Figure 3.**
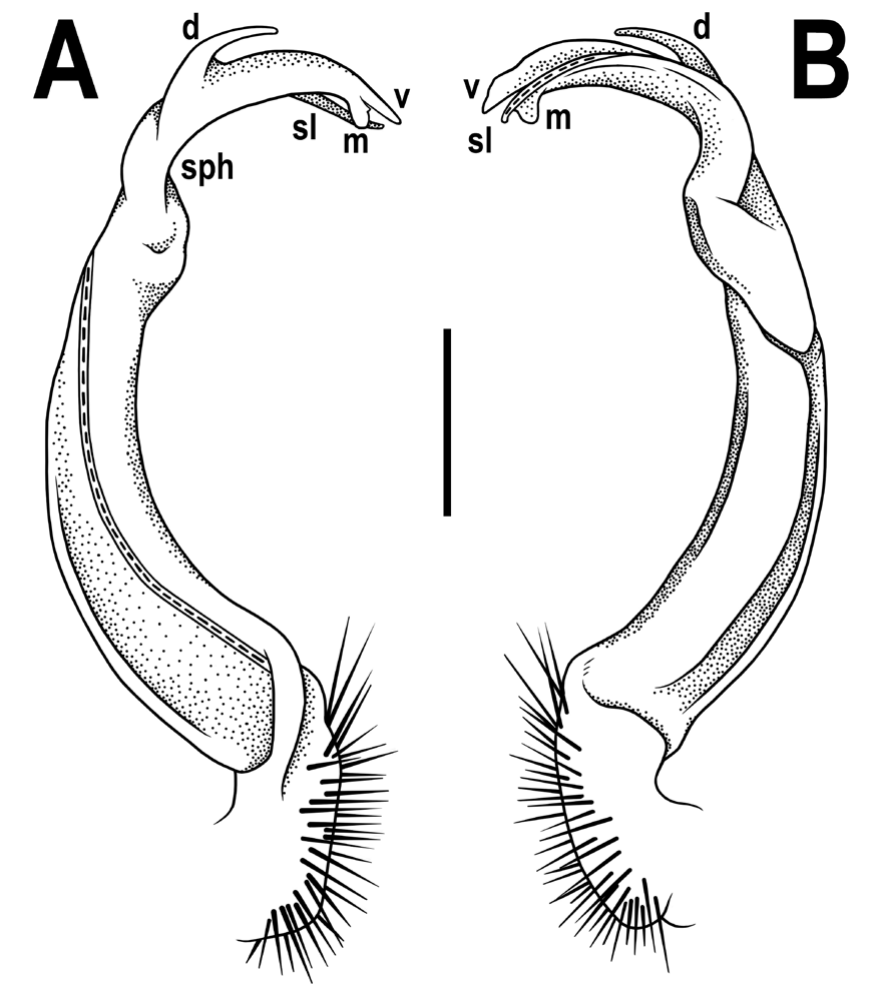
*Antheromorphanguyeni* sp. n., ♂ holotype. **A, B** left gonopod, mesal and lateral views, respectively. Scale bar: 0.5 mm.

**Figure 4.**
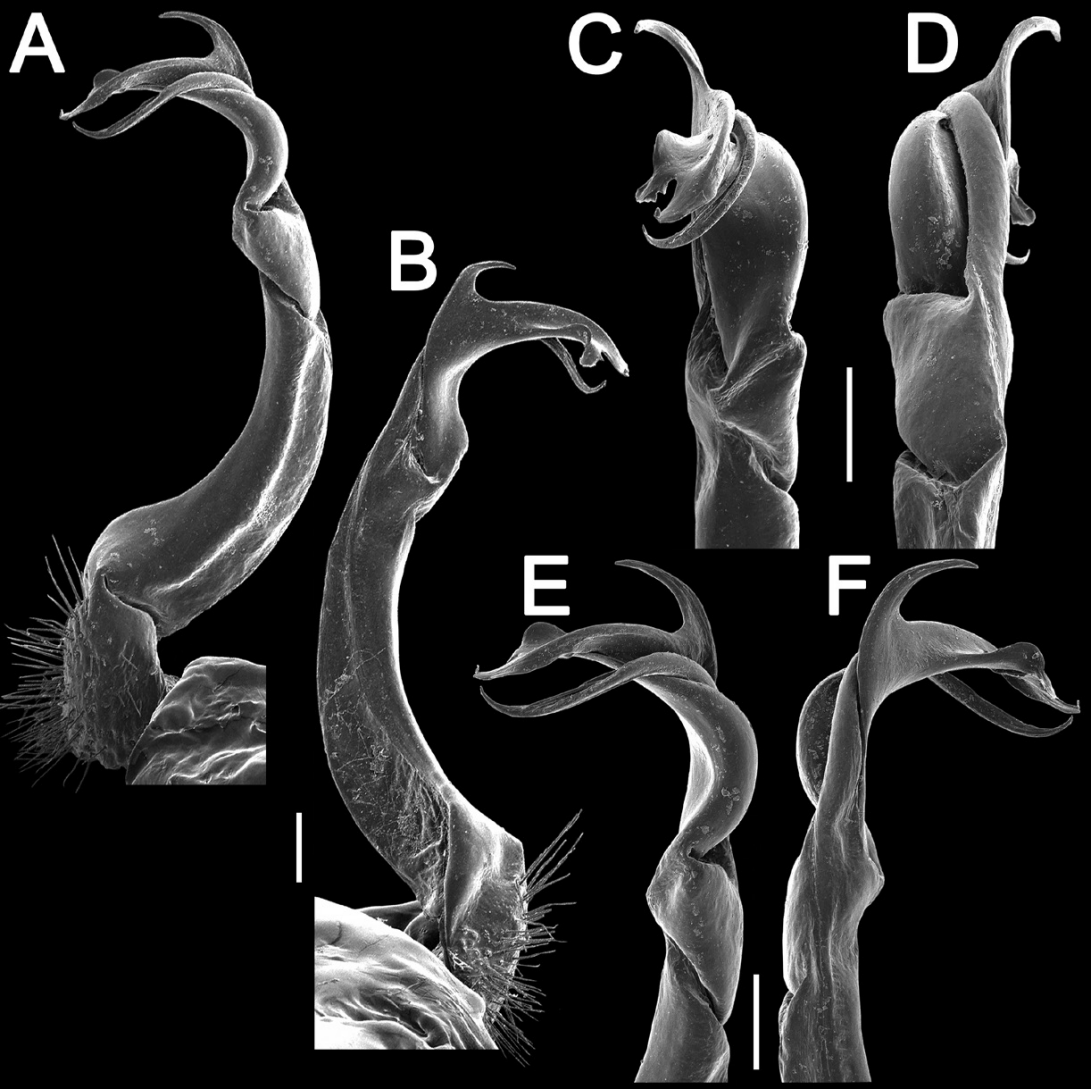
*Antheromorphanguyeni* sp. n., ♂ paratype. **A, B** left gonopod, lateral and mesal views, respectively **C–F** distal part of right gonopods, suboral, subcaudal, lateral and mesal views, respectively. Scale bar: 0.2 mm.

Gonopods (Figs 3, 4) simple, with femorite ca. 3 times as long as prefemoral (= strongly setose) part. Femorite moderately curved caudad, postfemoral portion demarcated by an oblique lateral sulcus; solenomere flagelliform, almost fully sheathed by solenophore, tip of solenophore very deeply bifid; with a rather long, slender, fully pointed process **d**; process **m** with a narrowly rounded terminal lobule, longer than a small, rounded process **v** with a very small, middle, spiniform prong.

######## Remarks.

Adults of the new species were found in May during a short expedition to a small area within Kon Ka Kinh National Park near its headquarters. A prospected forest with a similar forest structure within the same park near the village of Krong (N14°17', E108°26', 700–1000 m a.s.l.), ca. 14 km NE of the type locality, failed to reveal this species. It co-exists at the type locality together with *Orthomorphascabra* Jeekel, 1967 and three other *Orthomorpha* species, all apparently undescribed. According to IS’ observations, the five species share the same microhabitats. Given the conspicuously large and similar sizes of the adults of *Antheromorpha* and *Orthomorpha*, an ecological study of this syntopy in Kon Ka Kinh National Park would be worthwhile.

Although our new species is superficially very similar to *Orthomorpha* species such as *O.elevata* Likhitrakarn, Golovatch & Panha, 2011, from Perak State, Malaysia, it is clearly different in the shape of the sternal lamina between ♂ coxae 4 and in gonopodal structure (Likhitrakarn et al. 2011).

### Key to the known species of *Antheromorpha*, chiefly based on the male characters (modified mostly after Likhitrakarn et al. 2016)

**Table:** 

1	Colour pattern of metaterga: yellowish paramedian spots in front of transverse sulcus, the latter visible starting with segment 2	*** A. pardalis ***
–	Colour pattern of metaterga otherwise. Transverse sulcus present starting with segment 4 or 5	**2**
2	Colour pattern of metaterga: yellowish or brownish paramedian stripes	**3**
–	Colour pattern of metaterga otherwise	**9**
3	Gonopod femorite relatively short	**4**
–	Gonopod femorite long (Figs 3, 4A, B)	**6**
4	Midbody metazonae ca. 2.0 mm wide. Pleurosternal carinae poorly-developed, in ♂ slightly projecting caudad behind rear tergal margin only until segment 5	*** A. mediovirgata ***
–	Midbody metazonae ≥ 2.9 mm wide. Pleurosternal carinae well-developed, in ♂ slightly projecting caudad behind rear tergal margin at least until segment 10	**5**
5	Sternal lamina between ♂ coxae 4 with a paramedian pair of evident, high, nearly pointed, fully separated, setose cones. Gonopod process **d** very long	*** A. festiva ***
–	Sternal lamina between ♂ coxae 4 with only a single small cone. Gonopod process **d** shorter	*** A. bistriata ***
6	Sternal lamina between ♂ coxae 4 with a paramedian pair of separated lobes	**7**
–	Sternal lamina between ♂ coxae 4 a simple, rounded, conical knob	**8**
7	Paraterga narrow. Sternal lamina between ♂ coxae 4 a large, cordiform, ventrally evidently concave lobe. Gonopod solenophore clearly curved	*** A. comotti ***
–	Paraterga broad. Sternal lamina between ♂ coxae 4 a median pair of rather small and rounded lobes. Gonopod solenophore suberect and nearly straight	*** A. pumatensis ***
8	Metazonae ca. 2.6 mm (♂) or ca. 3.0 mm wide (♀). Gonopod femorite nearly straight, process **d** longer than solenophore	*** A. minlana ***
–	Metazonae 3.2–3.7 mm (♂) or 3.6–4.6 mm wide (♀). Gonopod femorite strongly curved caudad, process **d** shorter than solenophore	*** A. miranda ***
9	Sternum between ♂ coxae 4 with a single lamina or cone	**10**
–	Sternum between ♂ coxae 4 with a paramedian pair of separated cones	**11**
10	Colour pattern: a light axial stripe flanked on each side by a dark stripe on collum to epiproct. Epiproct simple, not particularly large, with two small, but evident apical papillae. Tip of gonopod split rather deeply, but process **d** shorter	*** A. uncinata ***
–	Colour pattern indistinct, with a pale yellowish median stripe against a uniformly brown background. Epiproct particularly large, with two apical papillae curved remarkably ventrad, claw-shaped. Tip of gonopod split deeper, process **d** very long	*** A. harpaga ***
11	Colour pattern: uniform with contrasting yellow or yellow-brown paraterga and epiproct (Fig. 1A–H). Gonopod process **d** simple, narrowly rounded apically (Figs 3, 4)	**12**
–	Colour pattern: a dark band present only on pinkish or pale yellowish posterior halves of proterga and posterior halves of metaterga. Gonopod process **d** clavate apically, rounded and significantly longer than process **m**	*** A. rosea ***
12	Sternum between ♂ coxae 4 with a pair of evident, high, separated cones. ♂ tarsal brushes present until legs of segment 8. Gonopod process **d** simple, as long as solenophore	*** A. paviei ***
–	Sternum between ♂ coxae 4 with a pair of small, low, separated cones. ♂ tarsal brushes present until segment 18. Gonopod process **d** very short, approx. half as long as solenophore	***A.nguyeni* sp. n.**

## Conclusions

**Figure 5.**
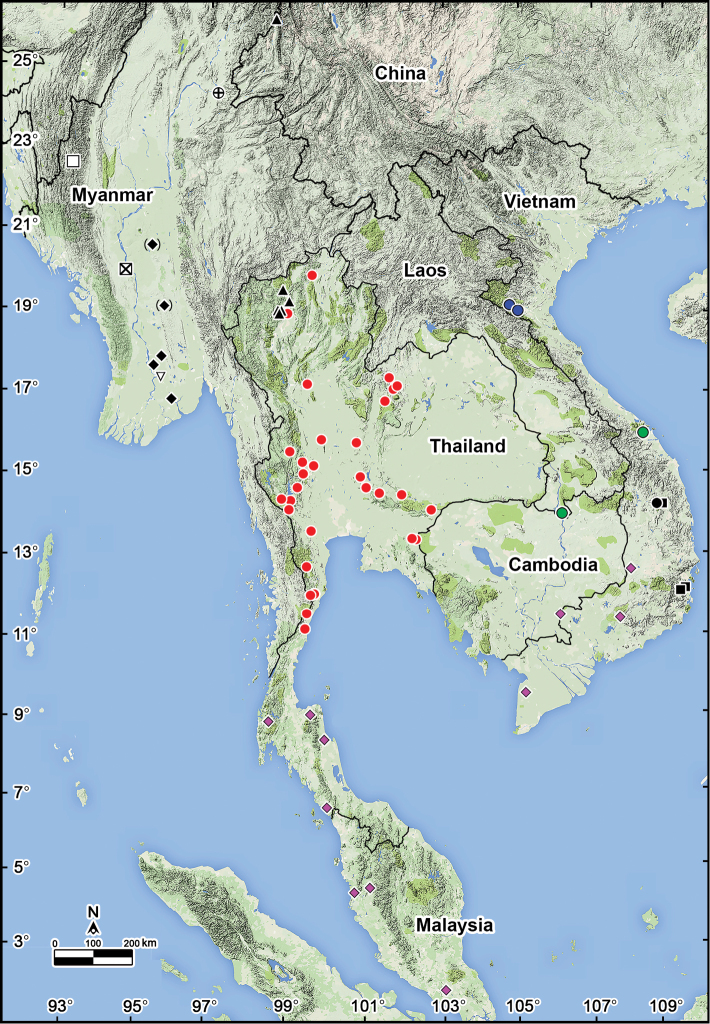
Distribution of *Antheromorpha* species (13) Key: Black triangle *A.rosea* Golovatch, 2013 Crossed circle *A.bistriata* (Pocock, 1895) Open square *A.comotti* (Pocock, 1895) and *A.mediovirgata* (Carl, 1941) Black diamond *A.miranda* (Pocock, 1895) Crossed square *A.comotti* (Pocock, 1895), *A.miranda* (Pocock, 1895) and *A.minlana* (Pocock, 1895) Inverted open triangle *A.pardalis* (Pocock, 1895) Red circle *A.uncinata* (Attems, 1931) Blue circle *A.pumatensis* Nguyen, Nguyen & Le, 2018 Green circle *A.paviei* (Brölemann, 1896) Black circle *A nguyeni* sp. n. Black square *A.harpaga* (Attems, 1938) Pink diamond *A.festiva* (Brölemann, 1896).

The genus *Antheromorpha* belongs to the tribe Orthomorphini which currently contains 25 genera (Nguyen and Sierwald 2013, Srisonchai et al. 2018a, b, c), this allocation being supported not only morphologically, but also by molecular evidence (Nguyen et al. 2018). *Antheromorpha* species range from southern China in the north, through Myanmar, Thailand, Laos, and Vietnam, to Western Malaysia in the south (Fig. 5). Four species seem to be particularly widespread; thus, *A.uncinata* occurs all over Thailand and it has repeatedly been reported swarming in the northern parts of the country (Likhitrakarn et al. 2016). A similarly vast distribution is also characteristic of *A.festiva*, which has been recorded across the southern half of the Malay Peninsula (southern Thailand and Western Malaysia) to southern Vietnam. As the range of *A.rosea* covers southern China and northern Thailand, most probably it occurs also in eastern Myanmar. Finally, since *A.paviei* is known from southern Laos and Vietnam, it seems very likely that it should exist in Cambodia as well (Likhitrakarn et al. 2014) (Fig. 5). Moreover, further *Antheromorpha* species may well be revealed with additional collecting efforts, especially in poorly prospected places so numerous in the huge region involved (Likhitrakarn et al. 2016).

## Supplementary Material

XML Treatment for Antheromorpha festiva

XML Treatment for Antheromorpha nguyeni
